# Unveiling pepper immunity’s robustness to temperature shifts: insights for empowering future crops

**DOI:** 10.1093/hr/uhae239

**Published:** 2024-08-21

**Authors:** William Billaud, Judith Hirsch, Valentin Ribaut, Lucie Tamisier, Anne Massire, Marion Szadkowski, Félicie Lopez-Lauri, Benoît Moury, Véronique Lefebvre

**Affiliations:** INRAE, GAFL, F-84140 Montfavet, France; INRAE, Pathologie Végétale, F-84140 Montfavet, France; Qualisud, Univ Montpellier, Avignon Univ, CIRAD, Institut Agro, Univ de La Réunion, Montpellier, France; INRAE, Pathologie Végétale, F-84140 Montfavet, France; INRAE, GAFL, F-84140 Montfavet, France; INRAE, Pathologie Végétale, F-84140 Montfavet, France; INRAE, GAFL, F-84140 Montfavet, France; INRAE, Pathologie Végétale, F-84140 Montfavet, France; INRAE, GAFL, F-84140 Montfavet, France; INRAE, Pathologie Végétale, F-84140 Montfavet, France; Qualisud, Univ Montpellier, Avignon Univ, CIRAD, Institut Agro, Univ de La Réunion, Montpellier, France; UPRI, ERIT Plant Science Interaction and Innovation, Avignon Université, Avignon, France; INRAE, Pathologie Végétale, F-84140 Montfavet, France; INRAE, GAFL, F-84140 Montfavet, France

## Abstract

Boosting plant immunity is an effective alternative to pesticides. However, environmental variations, accentuated by climate change, can compromise immunity. The robustness of a trait corresponds to the absence (or low level) of variation in that trait in the face of an environmental change. Here, we examined two types of robustness, robustness of immunity mean and robustness of immunity variation, and proposed nine quantitative robustness estimators. We characterized the immunity of a set of accessions representative of the natural diversity of pepper (*Capsicum annuum* L.), to two major pathogens: the oomycete *Phytophthora capsici* Leon. and potato virus Y. For each pathogen, we measured the immunity of accessions in two contrasting environments in terms of temperature. For each type of robustness and each pathogen, the impact of temperature change on immunity varied between accessions. The robustness estimators proved to be complementary and differed in terms of heritability and ability to discriminate accessions. A positive and significant correlation was observed between immunity and robustness. There was no significant relationship between the robustness of immunity to the two pathogens, but some accessions showed high immunity and robustness against both pathogens. These results justify the need to consider both immunity and robustness to environmental variations in order to select varieties adapted to current and future climate conditions. Phenotypic robustness should also be considered when assessing the “value of sustainable cultivation and use” of future plant varieties, particularly during the application process for protection rights granted from the European Community Plant Variety Office.

## Introduction

In their natural or agricultural environments, plants are exposed to a combination of biotic and abiotic perturbations. Many studies have shown that the environment and its variability, particularly temperature, impact plant immunity to pests and pathogens [1–5]. Variation of immunity is accentuated by the current context of climate change, which is responsible for increasing environmental variability. This includes the rise in average global temperature and the greater frequency and intensity of extreme climatic events [6]. Ensuring that plant immunity remains effective in different abiotic environments is therefore a major challenge for plant breeding to limit pathogen epidemics, reduce the use of pesticides and guarantee food safety.

**Figure 1:**
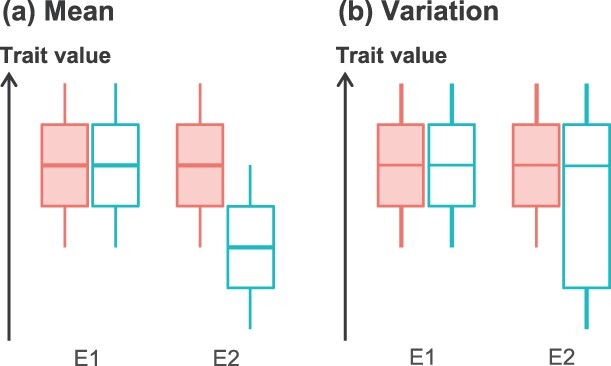
The concept of trait robustness. Robustness of the mean (**a**) and robustness of the variation (**b**) of a trait in two environments, E1 and E2. For each environment, the boxplots depict the mean (horizontal line) and the range of variation (height of the box) of this trait for two accessions, a robust one (filled) and a nonrobust one (empty).

The robustness of a phenotypic trait is defined by the absence or low level of variation of that trait in the face of a specific genetic or environmental perturbation [7]. The genetic perturbation can be a mutation, recombination, or hybridization; the environmental perturbation can be either biotic or abiotic. Félix and Barkoulas [7] distinguished two types of robustness corresponding to the stability of either the mean or the variation of a given trait in different environments, hereafter referred to, respectively, as robustness of the mean and robustness of the variation (Fig. 1). Various studies on the robustness of a trait in response to a perturbation have been conducted in plants [8–11]. Different methods have been proposed in the literature to estimate robustness or other associated traits such as phenotypic plasticity, but these methods have rarely been compared [7, 11–15]. Furthermore, the robustness of plant immunity has rarely been studied as a trait *per se* [7, 11].

In this article, we investigated the robustness of pepper immunity to temperature perturbation*.* Due to its nutritional value and economic importance, pepper is a major vegetable crop cultivated worldwide. It encounters a variety of climatic conditions that expose it to a wide range of pests and pathogens, resulting in significant yield losses. To ensure profitable harvests, the control of these pathogens heavily relies on the application of pesticides. Because the development of sustainable agriculture is a priority [16], pepper breeding has focused on developing resistant varieties [17]. Several genetic sources of immunity to pests and pathogens have been identified in pepper, but they have rarely been evaluated under multiple environmental conditions to study the fluctuation of immunity [18–26]. We measured the immunity of a core collection of *Capsicum annuum* L. to two major pathogens, under two contrasted temperature regimes: the oomycete *Phytophthora capsici* Leon., responsible for blight, fruit, and root rot, and potato virus Y (PVY), responsible for leaf mosaic and necrosis. From the immunity measures, we derived nine quantitative estimators of the robustness of immunity to the temperature change. We then examined the correlations between robustness estimators and immunity levels and compared their performance in terms of heritability and ability to discriminate accessions. Finally, we studied the genericity of immunity robustness by comparing the results obtained with the two pathogens.

## Results

### Temperature modulates the immunity of pepper accessions

To assess the effect of temperature change on the immunity of peppers to *P. capsici* and PVY, immunity was evaluated in two environments: a lower temperature regime (environment E1) specific to each pathogen and a 6°C higher temperature regime (environment E2). The susceptibility of 163 and 143 accessions *of C. annuum* to *P. capsici* and PVY, respectively, was assessed separately for each pathogen, by calculating the time-relative area under the disease progress curve for each plant, hereafter referred to as ${S}_{ijk}$, where *i* denotes the pepper accession, *j* the plant replicate, and *k* the environment. The susceptibility means ${S}_{i.k}$ of each accession in each environment were then calculated.

**Figure 2:**
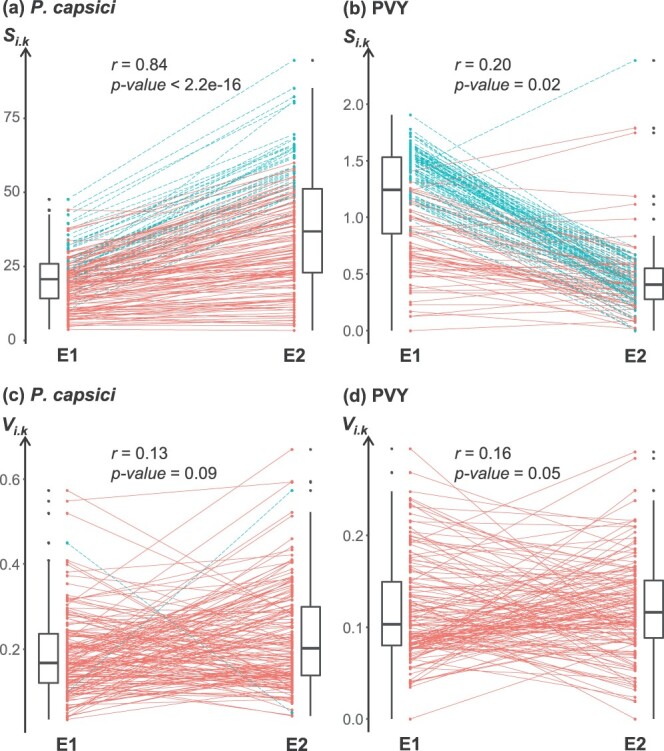
Immunity of a set of pepper accessions in two temperature environments. Susceptibility means ${S}_{i.k}$ to *Phytophthora capsici* (**a**) and to potato virus Y (PVY) (**b**). Susceptibility variations ${V}_{i.k}$ to *P. capsici* (**c**) and to PVY (**d**). Each dot represents ${S}_{i.k}$ or ${V}_{i.k}$ of accession *i* in environment *k*, and lines link the values of the same accession in the two environments. Dashed lines correspond to accessions that have a significantly different value in the two environments according to the TukeyHSD post hoc test (*p* < 0.05), while solid lines correspond to accessions that do not have significantly different values in the two environments. The correlation coefficients (Pearson’s *r*) between environments E1 and E2 and their *p*-values are shown on each graph.

Overall, temperature affected pepper immunity in opposite directions for the two pathogens. For *P. capsici*, the higher temperature environment almost doubled the overall susceptibility of the 163 accessions (${S}_{..E1}=20.5$ and ${S}_{..E2}=37.2$). A strong correlation between the susceptibility means ${S}_{i.k}$ was observed between the two environments (Pearson’s *r* = 0.84, *p* < 10^−16^, Fig. 2a). In contrast, for PVY, the higher temperature environment reduced the global susceptibility of the 143 accessions by about threefold (${S}_{..E1}=1.16$ and ${S}_{..E2}=0.44$), and a significant but weak correlation of susceptibility means ${S}_{i.k}$ was observed between the two environments (Pearson’s *r* = 0.20, *p* value = 0.02, Fig. 2b). Systemic leaf necrosis and plant death (corresponding to the highest symptom scores) were observed for some PVY-infected accessions in the lower temperature regime but only rarely in the higher temperature regime.

For both pathogens, the significant accession × environment interaction indicated that the effect of temperature on ${S}_{ijk}$ varied between accessions (Table S1). This interaction was less pronounced for *P. capsici* (percentage of phenotypic variation explained, PVE = 9.4%) than for PVY (PVE = 14.9%). This genotype-by-environment (G × E) interaction implies that the robustness of immunity varies between accessions, suggesting that it is genetically controlled. For both pathogens, some accessions showed little or no variation of ${S}_{i.k}$ between the two environments, whereas for other accessions, it differed greatly. No significant difference in ${S}_{i.k}$ between the two environments was observed for 125 of 163 accessions (77%) infected by *P. capsici*, nor for 58 of 143 accessions (41%) infected by PVY (Tukey's Honest Significant Difference _TukeyHSD_ post hoc tests, *p* > 0.05) (Fig. 2a, b).

For each pathogen, ${V}_{ijk}$, which expresses the deviation of ${S}_{ijk}$ from the susceptibility mean ${S}_{i.k}$ (see Materials and methods section) and the susceptibility variations ${V}_{i.k}$ of each accession in each environment were calculated. The significant accession effect on ${V}_{ijk}$ indicated that ${V}_{ijk}$ was genetically controlled. For both pathogens, the significant accession × environment interaction indicated that the effect of temperature on ${V}_{ijk}$ varied between accessions (Table S2). This interaction effect was similar for *P. capsici* and for PVY (PVE = 14.6% and 10.4%, respectively). However, the correlation between ${V}_{i.E1}$ and ${V}_{i.E2}$ assessed in environments E1 and E2 was weak (Pearson’s *r* ≤ 0.16) and not (or marginally for PVY) significant (Fig. 2c, d). No significant difference in ${V}_{i.k}$ between the two environments was observed for 161 of the 163 (99%) accessions infected with *P. capsici*, nor for all 143 accessions infected with PVY (TukeyHSD test, *p* > 0.05).

### Robustness of the mean and robustness of the variation are complementary traits

**Figure 3:**
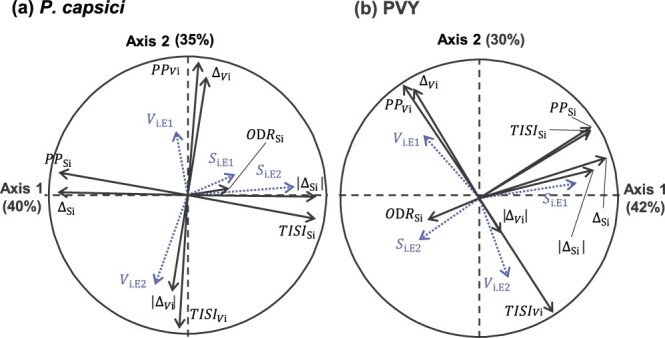
Principal component analyses of the estimators of robustness ${\Delta }_{S_i}$, $\left|{\Delta }_{S_i}\right|$, ${\mathrm{PP}}_{S_i}$, ${\mathrm{TISI}}_{S_i}$, ${\mathrm{ODR}}_{S_i}$, ${\Delta }_{V_i}$, $\left|{\Delta }_{V_i}\right|$, ${\mathrm{PP}}_{V_i}$, and ${\mathrm{TISI}}_{V_i}$, for *Phytophthora capsici* (**a**) and potato virus Y (PVY) (**b**). The susceptibility means ${S}_{i.k}$ and the susceptibility variations ${V}_{i.k}$ (dotted arrows) in environments E1 and E2 were added as illustrative variables.

For each pathogen, significant accession × environment interactions affected plant immunity, suggesting that the robustness of immunity varied between pepper accessions. To quantitatively characterize the robustness of each accession, we defined nine robustness estimators (see Materials and methods section). The estimators of the robustness of the mean are ${\Delta }_{S_i}$, $\left|{\Delta }_{S_i}\right|$, ${\mathrm{PP}}_{S_i}$, ${\mathrm{TISI}}_{S_i}$, and ${\mathrm{ODR}}_{S_i}$ based on the accession’s susceptibility mean ${S}_{i.k}$, and the estimators of the robustness of the variation are ${\Delta }_{V_i}$, $\left|{\Delta }_{V_i}\right|$, ${PP}_{V_i}$, and ${TISI}_{V_i}$, based on the accession’s susceptibility variation ${V}_{i.k}$. We compared these robustness estimators using principal component analyses (PCAs). For both pathogens, the first two axes of the PCAs explained more than 70% of the variation, and the robustness estimators were mainly distributed along these two axes (Figs 3**,**S1). The first axis primarily grouped estimators of the robustness of the mean (${\Delta }_{S_i}$, $\left|{\Delta }_{S_i}\right|$, ${\mathrm{PP}}_{S_i}$, and ${\mathrm{TISI}}_{S_i}$), which contributed almost equally to this axis. The second axis mainly grouped estimators of the robustness of the variation (${\Delta }_{V_i}$, ${\mathrm{PP}}_{V_i}$, and ${\mathrm{TISI}}_{V_i}$ for both pathogens, and $\left|{\Delta }_{V_i}\right|$ for *P. capsici* only), which contributed almost equally to this axis. For both pathogens, ${\mathrm{ODR}}_{S_i}$ was not significantly represented on the two main axes and therefore did not significantly contribute to explaining the variability of the data. The distinction between the two types of robustness was confirmed by the low pairwise correlations between estimators (Pearson’s |*r*| < 0.15 for *P. capsici* and |*r*| < 0.27 for PVY, Table S3). The accessions were distributed continuously along the PCA axes and no distinct groups of accessions could be distinguished (Fig. S1). Furthermore, none of the accessions made a particularly high contribution to either of the two axes (individual contributions of accessions ≤16% for *P. capsici* and ≤ 7% for PVY). Therefore, PCA results for both pathogens show a clear distinction between robustness of mean and robustness of variation and highlight a continuum of variation within the set of pepper accessions for both types of robustness. This is illustrated by a set of representative pepper accessions displaying contrasting levels of robustness of mean and robustness of variation (Fig. S2).

### Robustness estimators differ in their heritability and ability to discriminate accessions

**Figure 4:**
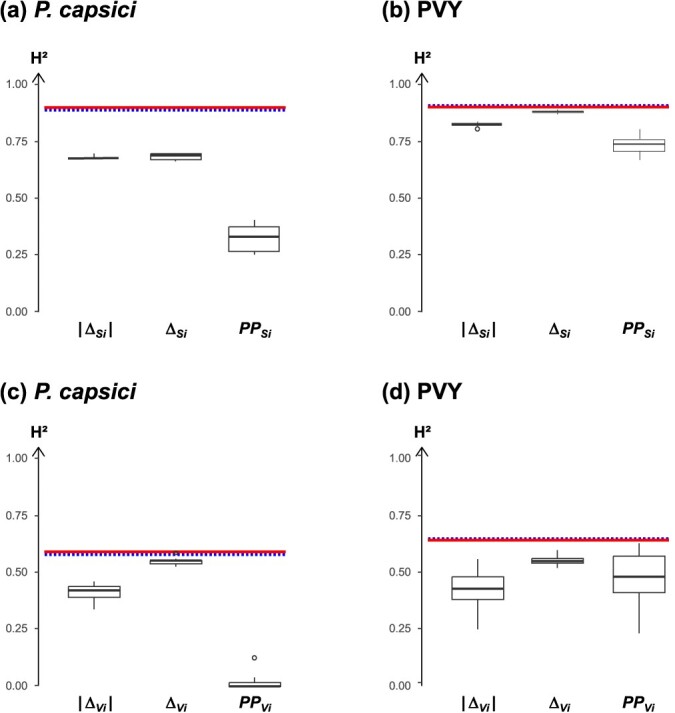
Broad-sense heritability of robustness estimators for immunity to *Phytophthora capsici* (**a**, **c**) and to potato virus Y (PVY) (**b**, **d**). Each boxplot corresponds to an estimator of the robustness of the mean (**a**, **b**) or variation (**c**, **d**). The boxplots were constructed from 100 *H*^2^ values obtained from 100 random pairs of data from the two environments for each accession. The dotted and solid lines correspond to the broad-sense heritability of the susceptibility mean ${S}_{i.k}$ (**a**, **b**) and the susceptibility variation ${V}_{i.k}$ (**c**, **d**) in environments E1 and E2, respectively. Heritability of $\mathrm{TISI}$ estimators could not be calculated because the random pairing method could not be applied (see Materials and methods section). Heritability of ${\mathrm{ODR}}_{Si}$ was omitted due to its poor ability to explain the variability observed in PCAs.

To evaluate the performance of the robustness estimators, we considered two metrics: (i) the broad-sense heritability (*H*^2^), which represents the proportion of the phenotypic variance explained by the genetic diversity between accessions and (ii) the discriminatory power, calculated as the percentage of pairs of accessions showing a significant difference in robustness based on TukeyHSD tests (%THSD). For both pathogens, ${\Delta }_{S_i}$ and $\left|{\Delta }_{S_i}\right|$ were the most heritable estimators of the robustness of the mean (mean H^2^ ≥ 0.67) and had the lowest range for their 100 estimated values (Fig. 4a, b). These values were close to the heritability of the susceptibility means ${S}_{i.E1}$ and ${S}_{i.E2}$ for both pathogens (H^2^ ≥ 0.89). Heritability was lower for ${\mathrm{PP}}_{Si}\kern0.5em$(mean H^2^ = 0.34 for *P. capsici* and 0.74 for PVY) with wider ranges of estimated values. With regards to the robustness of variation, ${\Delta }_{V_i}$ was the most heritable estimator for both pathogens (mean *H*^2^ ≥ 0.52) with the smallest ranges of estimated values (Fig. 4c, d). These values were close to the heritability of the susceptibility variation ${V}_{i.E1}$ and ${V}_{i.E2}$ for both pathogens (*H*^2^ = 0.58 to 0.62). Heritability of $\left|{\Delta }_{V_i}\right|$ was lower (mean H^2^ ≥ 0.38) and the range of estimated values was wider for both pathogens. Finally, ${\mathrm{PP}}_{V_i}$ showed contrasted heritability values depending on the pathogen. The heritability was moderate for PVY (mean *H*^2^ = 0.47) with a fairly wide range of estimated values, whereas it was very low for *P. capsici* (mean *H*^2^ ≤ 0.02).

**Figure 5:**
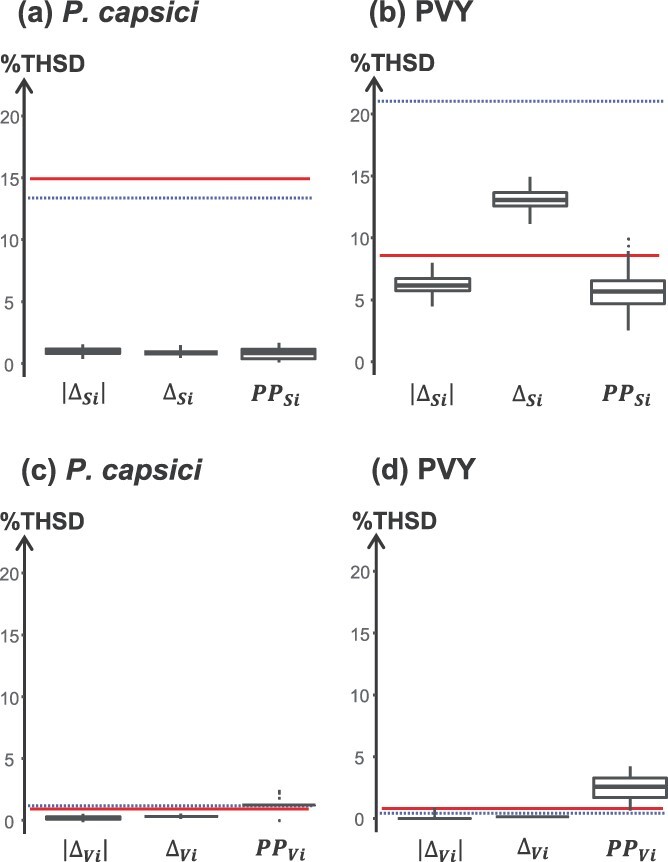
Discriminatory power (%THSD) of the robustness estimators for immunity to *Phytophthora capsici* (**a**, **c**) and to potato virus Y (PVY) (**b**, **d**). Each boxplot corresponds to an estimator of the robustness of the mean (**a**, **b**) or variation (**c**, **d**). The boxplots were constructed from 100 TukeyHSD values obtained from 100 random pairs of data from the two environments for each accession. The dotted and solid lines represent the discriminatory power of the susceptibility mean ${S}_{i.k}$ (**a**, **b**) and susceptibility variation ${V}_{i.k}$ (**c**, **d**) in environments E1 and E2, respectively.

For *P. capsici*, 13.4% and 14.9% of accession pairs had a significantly different susceptibility mean in the lower and higher temperature environments, respectively, while the discriminatory power of estimators of the robustness of the mean was much lower (%THSD ≤1%) (Fig. 5a). For PVY, 21.0% of accession pairs had a significantly different susceptibility mean in the low-temperature environment, whereas the discriminatory power was lower in the high-temperature environment (%THSD = 8.6%). The discriminatory power of estimators of the robustness of the mean ranged from 5.7% for ${\mathrm{PP}}_{S_i}$ to 13.1% for ${\Delta }_{S_i}$ (Fig. 5b). For both pathogens, the susceptibility variation ${V}_{i.k}$ and the estimators of the robustness of the variation had very low discriminatory power (mean %THSD ≤1.3% for *P. capsici* and ≤ 2.5% for PVY; Fig. 5c, d). This analysis highlights the low discriminatory power of the estimators of the robustness of the variation compared to the estimators of the robustness of the mean. In conclusion, ${\Delta }_{S_i}$, $\left|{\Delta }_{S_i}\right|$ and ${PP}_{S_i}$ were the most discriminating robustness estimators with variable performance depending on the pathogen. By considering heritability and the discriminatory power, ${\Delta }_{S_i}$ and $\left|{\Delta }_{S_i}\right|$ were the most efficient robustness estimators for *P. capsici*, while ${\Delta }_{S_i}$ and to a lower extent $\left|{\Delta }_{S_i}\right|$ and ${PP}_{S_i}$ were the most efficient robustness estimators for PVY.

### Significant correlation between immunity and robustness among accessions

For both pathogens, correlation coefficients between susceptibility means ${S}_{i.k}$ and robustness of the mean estimator $\left|{\Delta }_{S_i}\right|$ were significantly higher than expected under random permutations (*p*-values between 0.037 and < 0.001), except in one case: Pearson’s *r* correlation coefficient between susceptibility to PVY estimated in environment E2 and $\left|{\Delta }_{S_i}\right|$ (*p* value = 0.10; Table S4). This exception could be due to the lack of variation in susceptibility to PVY between accessions under the higher temperature regime, which does not allow a clear distinction between accessions (Fig. 2b). Apart from this particular case, immunity and robustness of the mean are therefore significantly correlated: the higher the immunity, the higher the robustness.

### The robustness of immunity is pathogen-specific

While the correlation between the accession susceptibility means ${S}_{i.k}$ to *P. capsici* and PVY was not significant in high-temperature environments, a significant but weak correlation (Pearson’s *r* = 0.23, *p*-value = 0.01) was observed in the low-temperature environments (Table S5a). Accordingly, no significant association was observed between the susceptibility mean (${S}_{i..}$, average of the two environments) to *P. capsici* and PVY when accessions were classified for each pathogen into two groups relative to the median of all accessions (Fisher’s exact test, *p*-value = 0.144, Table S5b).

The five estimators of the robustness of the mean of immunity were not or weakly correlated between *P. capsici* and PVY (|*r*| ≤ 0.22, Table S5c). There was no significant association between the robustness of the mean for immunity to the two pathogens (Fisher’s exact test, *p*-value = 0.835, Table S5d).

Regarding the susceptibility variation ${V}_{i.k}$ to the two pathogens, a significant but low correlation (*r* = 0.19, *p*-value = 0.03) was observed between *P. capsici* in the high-temperature environment and PVY in the low-temperature environment (Table S5e). No significant correlation between the susceptibility variation to the two pathogens was observed for the other combinations of environments. Additionally, no significant association was found between the susceptibility variation to the two pathogens after merging data from the two environments (Table S5f). Estimators of the robustness of the variation of immunity were not correlated between *P. capsici* and PVY (|*r*| ≤ 0.12, Table S5g).

Overall, our experimental results indicate that there is no strong association between the robustness of immunity to *P. capsici* and PVY, indicating that these traits are mostly genetically independent.

## Discussion

### A temperature rise of 6°C modulates pepper immunity differently depending on the pathogen

The robustness of agronomic traits is pivotal in plant breeding and agriculture, ensuring food safety under diverse environmental conditions [27]. Plant immunity to pathogens is profoundly impacted by abiotic perturbations, particularly temperature fluctuations [5, 28]. In most instances, high temperatures compromise plant immunity. Of the 38 pathosystems analyzed in 45 articles, 29 (76%) exhibited increased plant susceptibility or reduced plant defenses with rising temperatures, while only four pathosystems (10.5%) demonstrated the opposite trend [5]. This prompted us to investigate the impact of increasing temperature on the response of pepper to two major pathogens, *P. capsici* and PVY. We observed that a 6°C increase strongly altered the pepper immunity, albeit with contrasting effects on *P. capsici* and PVY: while immunity to *P. capsici* was nearly halved, immunity to PVY increased threefold on average across all pepper accessions. Importantly, this disparate effect underscores the specific nature of the influence of temperature on the plant–pathogen combination. It refutes the hypotheses of a universal suppression of pepper immunity by temperature rise or differences in pepper tolerance to high temperatures among accessions in the absence of pathogen infection. Previous studies have shown that pepper immunity to *P. capsici* is partially compromised at higher temperatures [22, 24, 29]. Furthermore, *in vitro* growth experiments revealed significantly accelerated growth of *P. capsici* at 30°C-day/28°C-night (environment E2) compared to 24°C day/22°C night (environment E1) (Fig. S3). Overall, in the case of *P. capsici*, these results suggest that temperature can have an impact on both plant immunity and the growth of micro-organisms. These findings align with the general observation that elevated temperatures exacerbate tissue necrosis and facilitate colonization by necrotrophic or hemi-biotrophic pathogens [30]. Regarding PVY, Tamisier *et al*. [31] proposed that systemic necrosis in pepper might be due to a hypersensitive response (HR), but triggered too slowly to impede virus infection. Indeed, genome-wide association studies identified a single QTL on pepper chromosome P9 associated with PVY-induced necrosis, colocalising with genes encoding receptor-like proteins or proteins belonging to the nucleotide-binding leucine-rich repeat (NLR) family. As high temperatures, approximately 30°C, are known to suppress HR expression in numerous pathosystems, systemic necrosis is expected be rare at high temperatures, which would explain the increased immunity to PVY.

In addition to the temperature effects on immunity, we observed moderate but highly significant genotype-by-environment (G × E) interactions (PVE = 9.4 to 14.9%, Table S1). This underscores the variability in the impact of temperature change on pepper immunity and, hence, the variability of the robustness of immunity in response to temperature change across accessions. Our subsequent challenge was to define quantitative estimators of robustness and compare them using our experimental datasets.

### Complementarity and performance of robustness estimators

The stability of phenotypic traits after a perturbation corresponds to various terms in biology, such as robustness [7] and canalization [32], and is opposed to phenotypic plasticity [12]. Although these terms have been adopted differently in various scientific disciplines (plasticity is widely used in ecology, while canalization and robustness are common in developmental biology and agronomy), they correspond to the same concept. Resilience, however, is a distinct concept referring to the capacity of a dynamic system to successfully respond to perturbations that threaten its viability, function, or development [33]. Resilience implies recovery —an intermediate state between the perturbation and the adapted state— when the system has been adversely affected, whereas robustness does not imply such an intermediate state. Furthermore, robustness can be estimated in various ways [12], but the different estimators have rarely been compared, especially in plant biology. Robustness is defined with regard to a specific phenotypic trait and a specific perturbation. In this respect, robustness differs from studies of the stability of phenotypic traits based, for example, on multisite or interannual trials, where the perturbation (or combination of perturbations) varies from one trial to another and is not always fully characterized [14].

Few studies have focused on the robustness of plant immunity in response to abiotic perturbations as a trait in its own right. Accordingly, little is known about the mechanisms underlying the robustness of immunity, their generality across different pathogens, or in response to different abiotic perturbations. Additionally, the relationship between the robustness of the mean and the robustness of the variation, as well as their connection to immunity itself, remains largely unexplored [7].

The robustness estimators we examined can be classified into three groups with low or no correlations between them (|*r*| ≤ 0.27 for *P. capsici*, |*r*| ≤ 0.38 for PVY): estimators of the robustness of the mean, estimators of the robustness of the variation, and the deviation from the orthogonal regression (${\mathrm{ODR}}_{S_i}$) estimator. In contrast, high correlations were generally observed between estimators within the same group (0.30 ≤ |*r*| ≤ 0.99 for both pathogens), except for the correlation between ${\boldsymbol{\Delta }}_{\boldsymbol{Vi}}$ and $\left|{\boldsymbol{\Delta }}_{\boldsymbol{Vi}}\right|$ for PVY (*r* = 0.01). Drawing on findings in animals and yeast, Mestek Boukhibar and Barkoulas [34] emphasized the nonredundancy and complementarity between robustness of mean and robustness of variation, highlighting the need to consider them separately when studying their distribution among genetic resources, the mechanisms involved, and their genetic determinants. Based on our results, we assume that the need to consider multiple estimators to study robustness is a fairly general result applicable to many plant species, agronomic or life-history traits, and pests and pathogens. We also suggest considering ${\mathrm{ODR}}_{Si}$ as a third type of robustness estimator for such analyses.

To extend our current work, it will be interesting to validate our findings across a broader range of temperatures through further experiments in more than two environments. This future research will involve using other robustness estimators, possibly inspired by the methods used in multisite studies to measure phenotypic stability (e.g. [14]).

Quantitative analyses confirmed that the robustness of the mean of immunity varied strongly and significantly between pepper accessions. In contrast, the variation of immunity (assessed here by the susceptibility variation) was rather low, regardless of the environment and the pathogen (Fig. 2c, d). This could be due to the highly homozygous status of the pepper accessions, which are reproduced by controlled self-pollination, and to the controlled environment of the assays. Consequently, few differences in ${V}_{i.k}$ were observed for accessions between environments, resulting in most of the accessions being considered robust to stochastic noise or micro-environmental variations. The low number of replicates per accession and per environment for each pathogen (6 and 10 plants inoculated with *P. capsici* and PVY, respectively) may also be responsible for a rather low statistical power, limiting the ability to detect significant differences in the robustness of the variation between accessions. Nevertheless, for both pathogens, ${V}_{i.k}$ varied significantly between accessions and the accession effect was similar (*p*-values < 10^−16^, PVE = 17.8% and 14.6% for *P. capsici* and PVY, respectively) (Table S2). We assume that ${V}_{i.k}$ reflects a stochastic process of symptom expression that differs between accessions. Indeed, Tamisier *et al*. [35] observed that different pepper accessions could impose a broader or narrower bottleneck at the inoculation step on virus populations, including PVY, and consequently different intensities of genetic drift and stochasticity on virus populations. Therefore, accessions imposing a greater genetic drift on pathogens could be responsible for greater variability of symptoms, thus a greater ${V}_{i.k}$.

The high to intermediate heritability estimates for most of the estimators of robustness of mean or variation indicate that the accession effect explains a large part of the variability in the robustness of immunity to *P. capsici* and PVY (Fig. 4). Lower heritability values for the robustness of variation could be due to a small number of plant replicates, affecting accurate estimation of immunity variation. This hypothesis may also explain the higher discriminatory power of the robustness of the mean compared to the robustness of the variation (Fig. 5). Despite the observed limitations for assessing the robustness of the variation, these findings demonstrate that the robustness of plant immunity to abiotic perturbations is genetically controlled, as evidenced in other studies on developmental or immunity traits in plants [11, 34]. The observed correlation between immunity and the robustness of immunity may be attributed to genes with pleiotropic effects governing both traits. Further investigation into the genetic determinism of these traits will enable us to test this hypothesis. This research paves the way for breeding varieties with a robust immunity to withstand environmental changes and for identifying key genetic factors underlying immunity robustness.

### Insights into the mechanisms and genericity of the immunity robustness

From an evolutionary perspective, organisms inhabiting fluctuating environments, considering either biotic or abiotic perturbations, may have acquired mechanisms to stabilize essential traits such as development, fertility, and immunity to various pests and pathogens. In *Drosophila* species, Rutherford and Lindquist [36] proposed that the chaperone Hsp90 plays a crucial role in controlling the robustness of several developmental traits. Queitsch *et al*. [37] further demonstrated that altering Hsp90 function in *Arabidopsis thaliana* resulted in a wide range of phenotype changes, supporting the central role of this protein in robustness. These observations raise questions about the genericity of robustness.

Our study reveals the relative independence of the robustness of immunity to the two pepper pathogens *P. capsici* and PVY (Table S5c, g), alongside the contrasting impact of a temperature change on immunity to these pathogens (Fig. 2). These findings suggest that the robustness of immunity to temperature changes is not inherently linked to the plant’s tolerance to temperature changes (in the absence of pathogens). We therefore propose that the effect of temperature on immunity primarily stems from its impact on the pathogen alone and/or on the interaction between the plant and pathogen, rather than on the plant alone. This indicates a lack of genericity of the robustness of immunity across different pathogens, suggesting that immunity robustness may be as specific as immunity itself.

Another question arises: does plant immunity robustness to a given pathogen remain effective across various environmental changes? In other words, is an immunity that is robust to a given temperature change also robust to other perturbations? The pepper accessions identified in this study, exhibiting contrasting levels of immunity robustness against *P. capsici* or PVY, constitute valuable experimental material for future investigations on this issue.

### Prospects for adapting crops to climate change

Although our study focused on the immunity of a single plant species to two specific pathogens, we consider that our results have generic relevance. These results highlight the existence of diverse robustness estimators for a specific trait and a specific perturbation. They also demonstrate that the robustness of immunity is specific to a given biotic perturbation, and highlight the independence between a trait, the nature of the perturbation, and its robustness. A more systematic analysis of robustness would therefore deserve to be extended, beyond immunity, to various traits of interest, in different species with respect to different biotic and abiotic perturbations. It could also encourage the development of varieties equipped with robust immunity or other robust traits across diverse environments, which would represent a significant advance for agriculture. Addressing how crop breeding can bolster sustainable production systems to mitigate the impacts of climate change, the European Commission has proposed in July 2023 a new regulation for testing and marketing varieties [38]. This proposal mandates that varieties must demonstrate a value for sustainable cultivation and use (VSCU) in addition to meeting the existing criteria for distinctness, uniformity, and stability. The primary challenge lies in evaluating the characteristics of plant varieties that contribute to sustainable production, including yield stability, disease resistance, climate change adaptation, and reduced dependence on pesticides and fertilizers. The conceptual framework proposed herein to assess trait robustness in varying environments can be utilized by breeders and regulatory bodies under the supervision of the Community Plant Variety Office, to establish the VSCU of a new variety. Our findings pinpoint promising genetic sources harboring both robust immunity to temperature changes and high-level immunity, thereby serving as potential sources of VSCU.

## Materials and methods

### Pepper immunity to *Phytophthora capsici*

**Figure 6:**
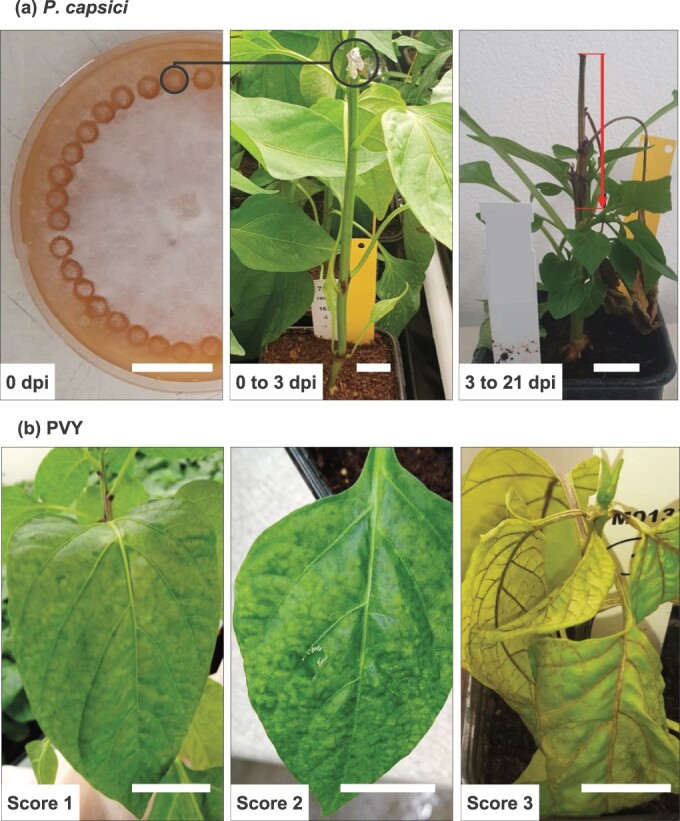
Assessment of pepper immunity to *Phytophthora capsici* and potato virus Y. (**a**) A V8-agar plug containing *P. capsici* mycelium was deposited on a decapitated plant and wrapped in aluminum foil. The length of necrosis induced by *P. capsici* development in the stem, indicated by the arrow, was measured at 3, 7, 10, 14, 17 and 21 days post-inoculation (dpi). (**b**) Systemic symptoms induced by potato virus Y on pepper leaves were assessed at 7, 14, 21, and 28 dpi. Score 0: absence of symptoms (not shown); score 1: weak symptoms; score 2: moderate symptoms; score 3: strong symptoms; score 4: plant death (not shown). The white horizontal bar corresponds to a length of 2 cm.

A set of 163 accessions from a *C. annuum* core collection [35] curated by the INRAE Centre for Vegetable Germplasm [39] were evaluated for their immunity to *P. capsici* in two distinct temperature environments: 24°C day/22°C night (environment E1) and 30°C day/28°C night (environment E2), each with a 12-hour photoperiod. These temperature conditions are known to elicit contrasted responses in immunity to *P. capsici* [22, 24]. A moderately aggressive *P. capsici* isolate, Pc107, was employed to maximize the range of the response to infection [40]. A mycelial plug (12 mm^2^) from the periphery of a V8 juice-agar medium Petri dish culture was deposited on the fresh stem section of 7-week-old plants and wrapped in 4 cm^2^ aluminium foil for three days. Necrotic lesion lengths along the stem were measured at 3, 7, 10, 14, 17, and 21 days post-inoculation (dpi), as described in [41] (Fig. 6a). In each environment, two independent assays were carried out, encompassing the entire process from sowing to inoculation and phenotyping. For each assay under each environment, three plants per accession were randomly distributed within the climate chamber.

### Pepper immunity to potato virus Y

A set of 143 accessions from the *C. annuum* core collection were evaluated for immunity to PVY. These accessions were chosen based on their ability to elicit a systemic necrotic reaction upon PVY inoculation under greenhouse conditions [31]. Of these accessions, 92 were common to those tested with *P. capsici*. Two distinct temperature environments were tested: 20°C day/18°C night (environment E1) and 26°C day/24°C night (environment E2), each with a 12-hour photoperiod. These temperature settings were based on preliminary experiments that demonstrated their contrasting effects on PVY immunity. Specifically, temperatures above 26°C resulted in high rates of non-infected plants, while temperatures below 18°C led to elevated plant mortality rates (unpublished data). Inoculation with PVY isolate SON41 was carried out mechanically by rubbing the inoculum onto the first fully developed leaf of 4-week-old plants grown in the greenhouse, as described in [42]. Systemic symptom severity was evaluated using a semi-quantitative scale ranging from 0 (no symptoms) to 4 (plant death) at 7, 14, 21, and 28 dpi (Fig. 6b). As with *P. capsici*, two independent assays were performed in each environment. For each assay under each environment, five plants per accession were randomized within the climate chamber. Plants showing no obvious viral symptoms were tested for PVY infection by DAS-ELISA on one gram of leaf tissue sampled on the uppermost fully expanded leaves at 28 dpi, as described in [43]. DAS-ELISAs were performed with a home-made polyclonal antiserum. We considered that samples with absorbance measured at 405 nm (A_405_) above a threshold of three times the mean A_405_ of noninfected controls were positive. Only PVY-positive plants were retained for further analysis to exclude plants that might have escaped infection.

### Assessment of pepper immunity levels and variation

To study the two types of robustness defined above (robustness of the mean and robustness of the variation of immunity; see Introduction section), we quantified both the mean and the variation of susceptibility for each pepper accession to each pathogen in each environment (E1 and E2), the term “susceptibility” being used here as the antonym of “immunity”. The susceptibility level ${S}_{ijk}$ was estimated for each plant using the time-relative area under the disease progress curve, which accounts for the possible death of the plant before the end of the assay [44]:


(1) \begin{equation*} {S}_{ijk}=\frac{\sum_{s=1}^{n_{ijk}-1\ }\frac{D_{ijk s}+{D}_{ijk\left(s+1\right)}}{2}\times \left({t}_{s+1}-{t}_s\right)}{T_{ijk}} \end{equation*}

where ${D}_{ijks}$ is the disease severity (necrosis length for *P. capsici*, symptom score for PVY) for plant replicate *j* of accession *i* in environment *k* (*k* = E1 or E2) at the *s*^th^ scoring, ${t}_s$ is the number of days after inoculation of the *s*^th^ scoring, ${n}_{ijk}$ is the total number of scorings, and ${T}_{ijk}$ is the number of days after inoculation of the last scoring (either at the end of the assay or at plant death).

The susceptibility variation ${V}_{ijk}$ was estimated for each plant by the mean-form of Levene’s statistic, which corresponds to the contribution of each plant to the within-accession variability [9, 12]:


(2) \begin{equation*} {V}_{ijk}=\left|\log{S}_{ijk}-\log{S}_{i.k}\right| \end{equation*}

where ${S}_{i.k}$ is the susceptibility mean of accession *i* in environment *k*.

### Factors influencing the mean and variation of immunity

For each pathogen, the effects of pepper accession, environment, and their interaction on susceptibility level and susceptibility variation were evaluated using analysis of variance (ANOVA). ANOVAs were performed using the “aov” function from the R package “stats”, using RStudio software (http://www.r-project.org/, RStudio 2022.07.1 Bild 554). Data from both assays were jointly analysed using the following fixed-effect model:


(3) \begin{align*} {Y}_{ijkt}=\mu& +{\mathrm{Acc}}_i+{\mathrm{Env}}_k+{\mathrm{Assay}}_t+\left({\mathrm{Acc}}_i\times{\mathrm{Env}}_k\right)+\left({\mathrm{Acc}}_i\times{\mathrm{Assay}}_t\right)\nonumber\\&+\left({\mathrm{Env}}_k\times{\mathrm{Assay}}_t\right)+\left({\mathrm{Acc}}_i\times{\mathrm{Env}}_k\times{\mathrm{Assay}}_t\right)+{\varepsilon}_{ijkt} \end{align*}

where ${Y}_{ijkt}$ is the susceptibility level ${S}_{ijkt}$ or the susceptibility variation ${V}_{ijkt}$ of plant replicate *j* from accession *i* in environment *k* in assay *t*, μ is the mean, ${\mathrm{Acc}}_i$ is the accession effect, ${\mathrm{Env}}_k$ is the environment effect, ${\mathrm{Assay}}_t$ is the assay effect. $\left({\mathrm{Acc}}_i\times{\mathrm{Env}}_k\right)$, $\left({\mathrm{Acc}}_i\times{\mathrm{Assay}}_t\right)$, $\left({\mathrm{Env}}_k\times{\mathrm{Assay}}_t\right)$, and $\left({\mathrm{Acc}}_i\times{\mathrm{Env}}_k\times{\mathrm{Assay}}_t\right)$ are the interaction effects between the factors, and ${\varepsilon}_{ijkt}$ is the residual. For each pathogen, data from both assays were also analysed separately using the following fixed-effect model:


(4) \begin{equation*} {Y}_{ijk}=\mu +{\mathrm{Acc}}_i+{\mathrm{Env}}_k+\left({\mathrm{Acc}}_i\times{\mathrm{Env}}_k\right)+{\varepsilon}_{ijk} \end{equation*}

where ${Y}_{ijk}$ is the susceptibility level ${S}_{ijk}$ or the susceptibility variation ${V}_{ijk}$, and ${\varepsilon}_{ijk}$ is the residual.

A Tukey's Honest Significant Difference post hoc test (TukeyHSD) implemented in the “TukeyHSD” function of the R package “stats” was applied with a *p-*value threshold of 0.05 to distinguish accessions with a significant phenotypic difference between environments.

### Estimators of robustness: $\Delta$, $\left|\Delta \right|$, $\mathrm{PP}$, $\mathrm{ODR}$, and $\mathrm{TISI}$

For each pepper accession confronted to a specific pathogen, we derived five estimators of the robustness of the mean, based on the susceptibility mean ${S}_{i.k}$ of accession *i* in environment *k*.

(i) ${\Delta }_{Si}={S}_{i.E1}-{S}_{i.E2}$ is the difference between the susceptibility means of accession *i* in environments E1 and E2.

(ii) $\left|{\Delta }_{Si}\right|$ is the absolute value of ${\Delta }_{Si}$ [12].

(iii) ${\mathrm{PP}}_{Si}=\frac{\left({S}_{i.E1}-{S}_{i.E2}\right)}{S_{i.E1}}$ represents the phenotypic plasticity of the susceptibility mean of accession *i* [45], indicating the gain (when ${\mathrm{PP}}_{Si}>0$) or loss (${\mathrm{PP}}_{Si}<0$) of immunity in environment E2 compared to environment E1.

(iv) ${\mathrm{ODR}}_{Si}$ is the orthogonal distance regression, measured as the orthogonal distance between the point corresponding to the two susceptibility means Si.E1 and Si.E2 for accession i and the regression line between the ${S}_{i.E1}$ and ${S}_{i.E2}$ values for all accessions, using the “odregress” function of the “pracma” R package.

(v) ${\mathrm{TISI}}_{Si}=\frac{1-\frac{{\mathrm{LSM}}_{S_{i.E2}}}{{\mathrm{LSM}}_{S_{i.E1}}}}{1-\frac{{\mathrm{LSM}}_{S_{..E2}}}{{\mathrm{LSM}}_{S_{..E1}}}}$ is the temperature-induced susceptibility index [11], where ${\mathrm{LSM}}_{S_{i.E1}}$and ${\mathrm{LSM}}_{S_{i.E2}}$are the least-squares means of the susceptibility mean ${S}_{i.k}$ of accession *i* in environments E1 and E2, respectively, and ${\mathrm{LSM}}_{S_{..E1}}\kern0.5em$and ${\mathrm{LSM}}_{S_{..E2}}$are the least-squares means of the susceptibility mean among all accessions in environments E1 and E2, respectively. The least-squares means were calculated from the fixed-effect model (4) using the “emmeans” function from the “emmeans” R package.

The closer ${\Delta }_{Si}$ or ${\mathrm{PP}}_{Si}$ to zero, the more robust the accession. The smaller $\left|{\boldsymbol{\Delta }}_{\boldsymbol{Si}}\right|$, ${\mathrm{ODR}}_{Si}$, or ${\mathrm{TISI}}_{Si}$, the more robust the accession.

Using the mean susceptibility variation ${V}_{i.k}$ of accession *i* in environment *k*, we derived four estimators of the robustness of the variation, calculated similarly to those developed for the robustness of the mean: ${\Delta }_{Vi}$, $\left|{\Delta }_{Vi}\right|$, ${\mathrm{PP}}_{Vi}$, and ${\mathrm{TISI}}_{Vi}$. Due to the low correlation between ${V}_{i.E1}$ and ${V}_{i.E2}$ for both pathogens, we considered it irrelevant to use the deviation from orthogonal regression ${\mathrm{ODR}}_{Vi}$ as an estimator of the robustness of the variation.

These calculations yielded a total of nine robustness estimators per accession and per pathogen.

### Complementarity of immunity traits and robustness estimators

We performed PCAs of the nine robustness estimators using the “PCAshiny” function of the “FactoShiny” R package. To identify relevant components for analyses and the variables with significant contributions to each component, we compared the PCA results with those obtained from 1000 PCAs performed on randomly permuted data, using the three statistics recommended by Björklund [46]. The susceptibility means ${S}_{i.k}$ and susceptibility variations ${V}_{i.k}$ in the two environments were included as illustrative variables in the PCAs.

For each pathogen, Pearson's correlation coefficients (*r*) between the four immunity traits ${S}_{i.k}$ and ${V}_{i.k}$ or between the nine robustness estimators were calculated using the “cor.test” function of the “stats” R package. Robustness estimators are derived from immunity measures, making immunity and robustness dependent variables. Consequently, correlations between immunity and robustness are likely to be spurious [47]. To circumvent this risk, we compared the correlation coefficients obtained from real datasets with those from randomly permuted datasets [48]. ${S}_{i.E1}$ (or ${S}_{i.E2}$) values were randomly permuted between accessions, and $\left|{\Delta }_{S_i}\right|$calculated. We then computed the correlation coefficient between ${S}_{i.E1}$ (or ${S}_{i.E2}$) and $\left|{\Delta }_{S_i}\right|$, using 1000 randomly permuted datasets for each pathogen and each susceptibility estimate.

Correlation coefficients of susceptibility mean, susceptibility variation and robustness estimators were also calculated between PVY and *P. capsici*. Additionally, for ${S}_{i..}$, ${V}_{i..}$, and $\left|{\Delta }_{S_i}\right|$, accessions were classified into two categories depending on whether their value was above or below the median of all accessions. Fisher’s exact tests were performed with the "fisher.test" function of the “stats” R package to test for a significant association of these variables between the two pathogens.

### Performance of immunity traits and robustness estimators

Since the robustness estimators were calculated from the susceptibility mean ${S}_{i.k}$ and the susceptibility variation ${V}_{i.k}$ for each accession *i* in environments E1 and E2, we can only derive a single robustness value for each accession. However, as the estimation of the trait heritability requires several independent values for each accession, we generated for each accession a set of independent robustness values by randomly pairing susceptibility values from one plant in environment E1 and one plant in environment E2. To ensure independence between the pairs, each susceptibility value was used only once in the pairings. We performed 100 random pairing simulations to obtain 100 heritability values per robustness estimator in order to estimate the mean heritability and its range. The random pairing method could not be applied to the ${\mathrm{TISI}}_{\mathrm{Si}}$ and ${\mathrm{TISI}}_{\mathrm{Vi}}$ estimators, as their calculation implies the calculation of a least-squares mean value per accession per environment, which does not permit the random pairing of susceptibility values ${S}_{ijk}$ or ${V}_{ijk}$ at the plant level. Additionally, the heritability of ${\mathrm{ODR}}_{\mathrm{Si}}$ has not been calculated because this estimator does not explain the variability observed.

The broad-sense heritability (H^2^) of the susceptibility mean, susceptibility variation, and the six remaining robustness estimators was calculated using the “repeatability” function from the R package “heritability” with the following formula:


(5) \begin{equation*} {H}^2=\frac{\sigma ^{2}_g}{\sigma ^{2}_g+\frac{\sigma ^{2}_e}{r}} \end{equation*}

where $\mathrm{\sigma} ^{2}_{\mathrm{g}}$ is the genetic variance, $\mathrm{\sigma} ^{2}_{\mathrm{e}}$ is the residual variance, and *r* is the number of plant replicates per accession.

To compare the ability of the six estimators of robustness to discriminate accessions, we applied a TukeyHSD test to the 100 robustness values obtained by random pairing. The TukeyHSD test determined for each pair of accessions whether their robustness was significantly different (*p-*value < 0.05). For each of the 100 random pairings, the discriminatory power of a robustness estimator was estimated by the percentage of pairs of accessions with significantly different robustness values (%THSD).

## Supplementary Material

Web_Material_uhae239

## Data Availability

The dataset and code generated for this study are accessible at https://doi.org/10.57745/3MN2BU [49].
