# Supplementary figures and images for: Unveiling pepper immunity’s robustness to temperature shifts: insights for empowering future crops
Source: Hortic Res. 2024 Aug 21;11(11):uhae239. doi: 10.1093/hr/uhae239 (PMC11540760; doi:10.1093/hr/uhae239)

Figure S1

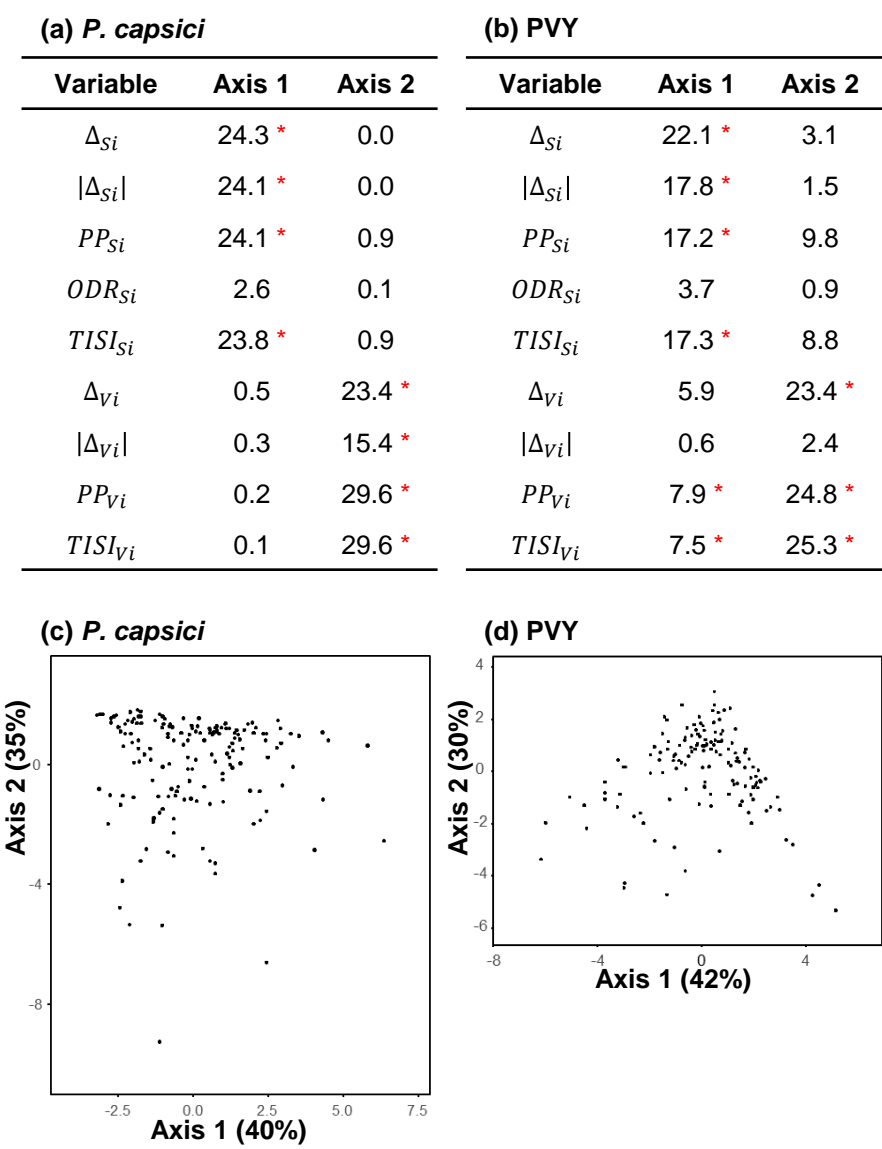

Supplement: Web_Material_uhae239 [file web_material_uhae239.zip › FigureS1_revised.pdf]

Figure S2

(a) *P. capsici*

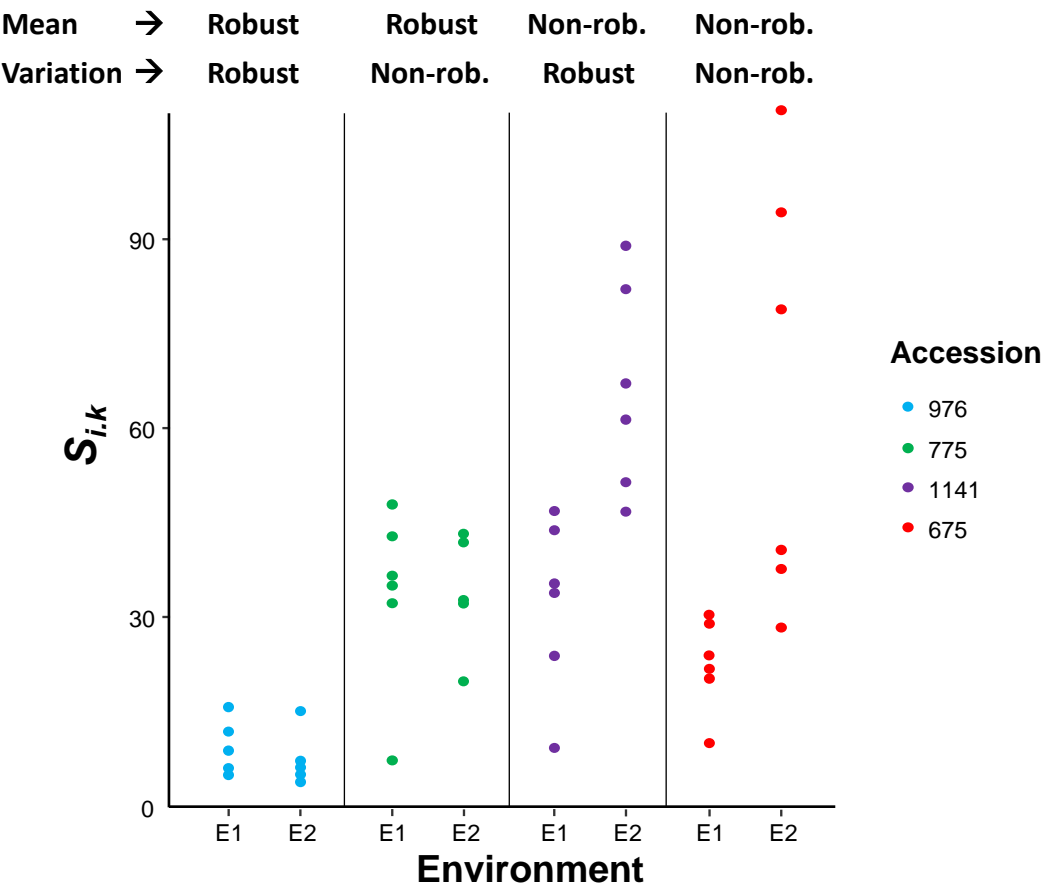

(b) Potato virus Y

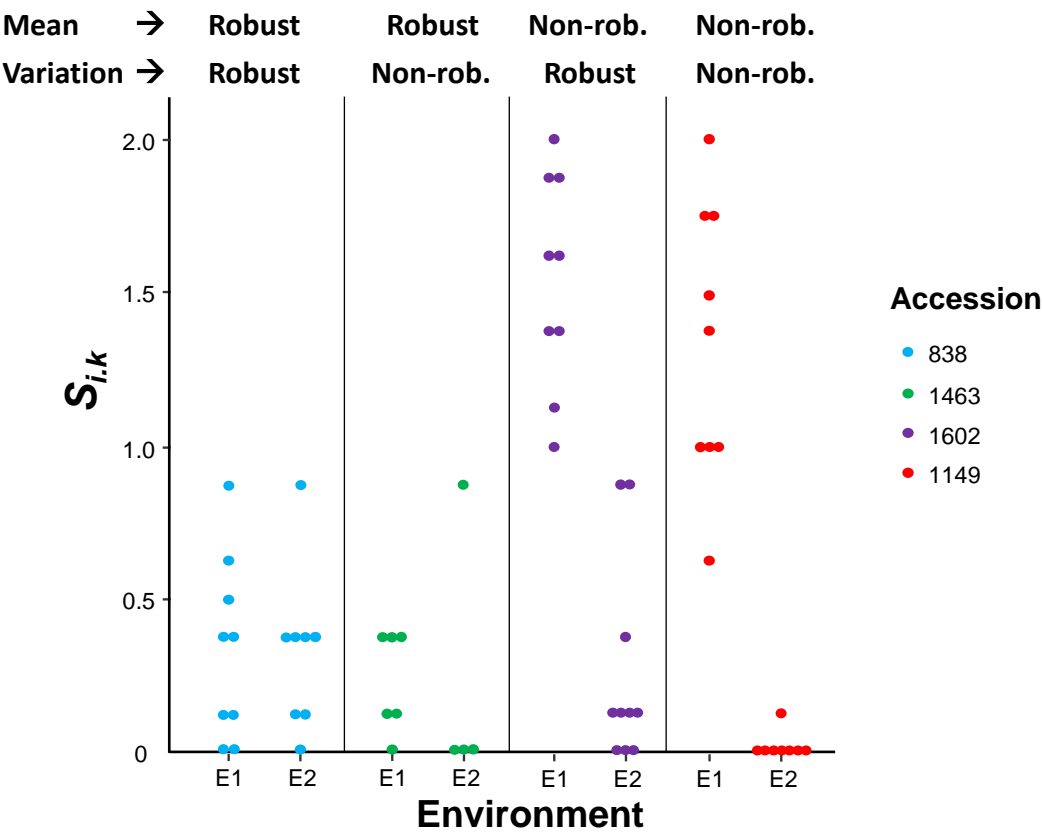

Supplement: Web_Material_uhae239 [file web_material_uhae239.zip › FigureS2_revised.pdf]

Figure S3

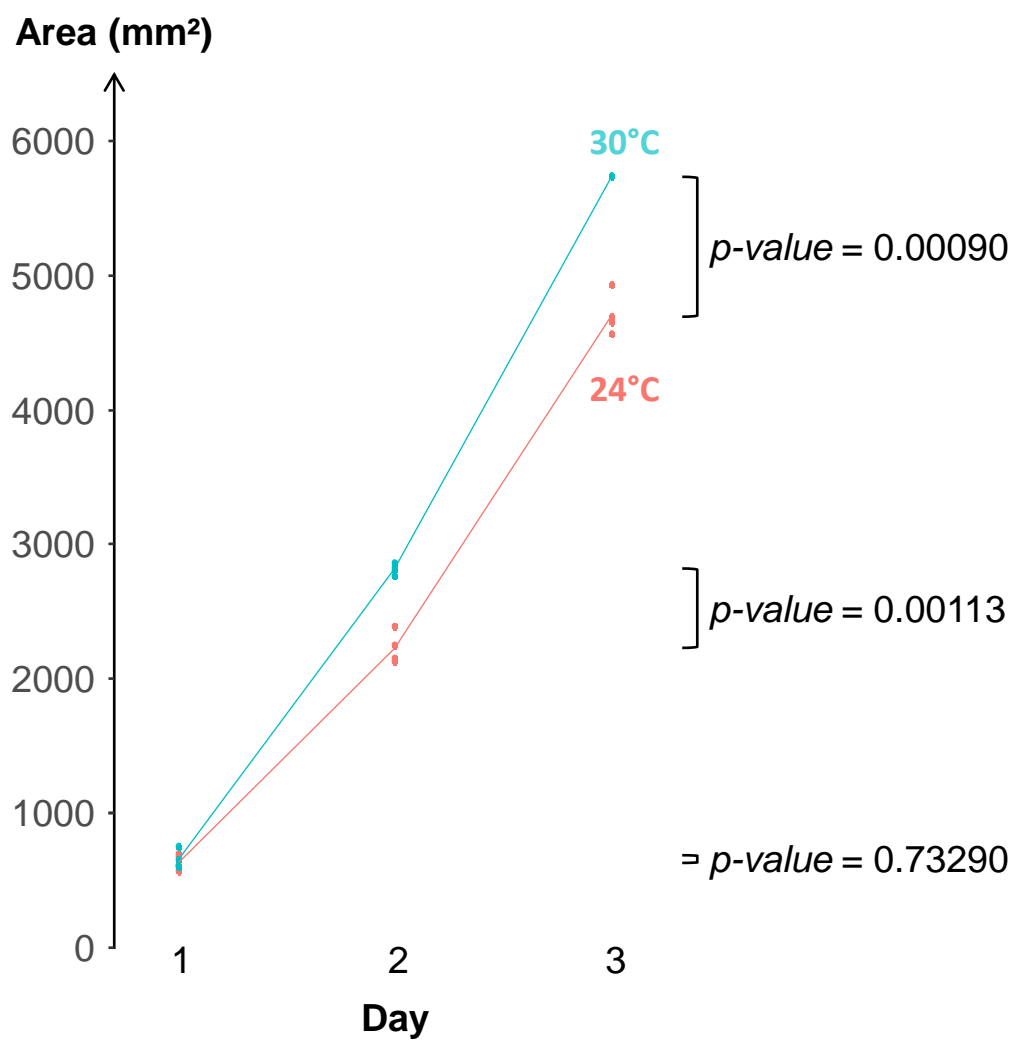

Supplement: Web_Material_uhae239 [file web_material_uhae239.zip › FigureS3_revised.pdf]
